# RETRACTION: LINC01614: A Potential Therapeutic Target in Astrocytoma Progression

**DOI:** 10.1111/jcmm.71311

**Published:** 2026-08-12

## Abstract

**RETRACTION**: F. Karimpour, M. H. Hooshiar, S. Abbasnejad, K. Abdi, A. Jalili, A. Khanmirzaei, S. Tutunchi, A.‐R. Javanmard, M. Hajiesmaeili, S. M. H. Ghaderian, “LINC01614: A Potential Therapeutic Target in Astrocytoma Progression,” *Journal of Cellular and Molecular Medicine* 29, no. 15 (2025): e70623. https://doi.org/10.1111/jcmm.70623.

The above article, published online on 30 July 2025 in Wiley Online Library (wileyonlinelibrary.com), has been retracted by agreement between the journal Editor‐in‐Chief, Stefan Constantinescu; and John Wiley & Sons Ltd. A third party reported on PubPeer [1] that the ethics approval numbers listed in this article (IR.SBMU.RETECH.REC.1398.485 and IR.SBMU.RETECH.REC.1399.1330) do not correspond to the topic of the article. Further reports from another third party expressed concerns that the western blot experiments reported in Figure 3 did not specify target proteins, lacked controls, and did not contain sufficient methodological details. The editors reviewed these concerns and agreed that the Methods presented in the article contained insufficient information.

Additional investigation by the publisher found that the Mock+PBS data in Figure 6A and Knock down Linc01614 data in Figure 6B had also been published in another article by a different author group [Liu et al. 2025 (https://doi.org/10.1007/s12672‐025‐02748‐0)]. Both articles were under review at their respective journals at the same time and both articles report these data as deriving from different experimental conditions. The investigation also found that Supplementary File S1 did not correlate to its in‐text citation.

The authors responded to an inquiry by the publisher and stated that both ethical approval codes were supplied because the research in this article used samples collected from two other clinical projects involving patients with diagnosed glioma and astrocytoma cases. They further stated that the apoptosis assay data were generated by an external service provider and that similarity between the data was coincidental. Original data presented in Figure 6 were provided by the authors. Subsequently, a review by an independent expert found that the data and explanation were insufficient and that there were discrepancies between the data provided and the results presented in the article.

The publisher and journal editorial board agree that they cannot verify the concerns regarding ethical approval. However, while the authors provided some further information regarding the methods and the data issues, their response was not adequate to address these concerns. The retraction has been agreed to by the publisher and editorial board because the evidence of data re‐use with another article, combined with concerns about the reliability of the methods and further concerns about the original data, fundamentally compromises the editors’ confidence in the results presented. The authors were informed of the retraction.

Reference

[1] Saccharothrix carnea . Comments on “LINC01614: A Potential Therapeutic Target in Astrocytoma Progression,” PubPeer, October 2025‐November 2025. https://pubpeer.com/publications/5508EC34FF73496C0ED0266353E94F.

**RETRACTION**: F. Karimpour, M. H. Hooshiar, S. Abbasnejad, K. Abdi, A. Jalili, A. Khanmirzaei, S. Tutunchi, A.‐R. Javanmard, M. Hajiesmaeili, S. M. H. Ghaderian, “LINC01614: A Potential Therapeutic Target in Astrocytoma Progression,” *Journal of Cellular and Molecular Medicine* 29, no. 15 (2025): e70623. https://doi.org/10.1111/jcmm.70623.

The above article, published online on 30 July 2025 in Wiley Online Library (wileyonlinelibrary.com), has been retracted by agreement between the journal Editor‐in‐Chief, Stefan Constantinescu; and John Wiley & Sons Ltd. A third party reported on PubPeer [1] that the ethics approval numbers listed in this article (IR.SBMU.RETECH.REC.1398.485 and IR.SBMU.RETECH.REC.1399.1330) do not correspond to the topic of the article. Further reports from another third party expressed concerns that the western blot experiments reported in Figure 3 did not specify target proteins, lacked controls, and did not contain sufficient methodological details. The editors reviewed these concerns and agreed that the Methods presented in the article contained insufficient information.

Additional investigation by the publisher found that the Mock+PBS data in Figure 6A and Knock down Linc01614 data in Figure 6B had also been published in another article by a different author group [Liu et al. 2025 (https://doi.org/10.1007/s12672‐025‐02748‐0)]. Both articles were under review at their respective journals at the same time and both articles report these data as deriving from different experimental conditions. The investigation also found that Supplementary File S1 did not correlate to its in‐text citation.

The authors responded to an inquiry by the publisher and stated that both ethical approval codes were supplied because the research in this article used samples collected from two other clinical projects involving patients with diagnosed glioma and astrocytoma cases. They further stated that the apoptosis assay data were generated by an external service provider and that similarity between the data was coincidental. Original data presented in Figure 6 were provided by the authors. Subsequently, a review by an independent expert found that the data and explanation were insufficient and that there were discrepancies between the data provided and the results presented in the article.

The publisher and journal editorial board agree that they cannot verify the concerns regarding ethical approval. However, while the authors provided some further information regarding the methods and the data issues, their response was not adequate to address these concerns. The retraction has been agreed to by the publisher and editorial board because the evidence of data re‐use with another article, combined with concerns about the reliability of the methods and further concerns about the original data, fundamentally compromises the editors’ confidence in the results presented. The authors were informed of the retraction.

**Reference**

[1] Saccharothrix carnea . Comments on “LINC01614: A Potential Therapeutic Target in Astrocytoma Progression,” PubPeer, October 2025‐November 2025. https://pubpeer.com/publications/5508EC34FF73496C0ED0266353E94F.

